# Mechanical Stretch Inhibits MicroRNA499 via p53 to Regulate Calcineurin-A Expression in Rat Cardiomyocytes

**DOI:** 10.1371/journal.pone.0148683

**Published:** 2016-02-09

**Authors:** Su-Kiat Chua, Bao-Wei Wang, Li-Ming Lien, Huey-Ming Lo, Chiung-Zuan Chiu, Kou-Gi Shyu

**Affiliations:** 1 Graduate Institute of Clinical Medicine, College of Medicine, Taipei Medical University, Taipei, Taiwan; 2 Division of Cardiology, Department of Internal Medicine, Shin Kong Wu Ho-Su Memorial Hospital, Taipei, Taiwan; 3 School of Medicine, Fu-Jen Catholic University, Taipei County, Taiwan; 4 School of Medicine, College of Medicine, Taipei Medical University, Taipei, Taiwan; 5 Department of Neurology, Shin Kong Wu Ho-Su Memorial Hospital, Taipei, Taiwan; Harbin Medical University, CHINA

## Abstract

**Background:**

MicroRNAs play an important role in cardiac remodeling. MicroRNA 499 (miR499) is highly enriched in cardiomyocytes and targets the gene for Calcineurin A (CnA), which is associated with mitochondrial fission and apoptosis. The mechanism regulating miR499 in stretched cardiomyocytes and in volume overloaded heart is unclear. We sought to investigate the mechanism regulating miR499 and CnA in stretched cardiomyocytes and in volume overload-induced heart failure.

**Methods & Results:**

Rat cardiomyocytes grown on a flexible membrane base were stretched via vacuum to 20% of maximum elongation at 60 cycles/min. An in vivo model of volume overload with aorta-caval shunt in adult rats was used to study miR499 expression. Mechanical stretch downregulated miR499 expression, and enhanced the expression of CnA protein and mRNA after 12 hours of stretch. Expression of CnA and calcineurin activity was suppressed with miR499 overexpression; whereas, expression of dephosphorylated dynamin-related protein 1 (Drp1) was suppressed with miR499 overexpression and CnA siRNA. Adding p53 siRNA reversed the downregulation of miR499 when stretched. A gel shift assay and promoter-activity assay demonstrated that stretch increased p53 DNA binding activity but decreased miR499 promoter activity. When the miR499 promoter p53-binding site was mutated, the inhibition of miR499 promoter activity with stretch was reversed. The in vivo aorta-caval shunt also showed downregulated myocardial miR499 and overexpression of miR499 suppressed CnA and cellular apoptosis.

**Conclusion:**

The miR499-controlled apoptotic pathway involving CnA and Drp1 in stretched cardiomyocytes may be regulated by p53 through the transcriptional regulation of miR499.

## Introduction

MicroRNAs (miRNA) are approximately 22 nucleotides long, non-coding RNAs that act as negative regulators of gene expression by interacting with the 3’-untranslated regions of target mRNA and promoting mRNA degradation (gene silencing).[1] A single miRNA can modulate complex physiological phenotypes by regulating cardiac function, including electrical signal conduction and cardiomyocyte contraction and growth.[2–5] Global miRNA expression profiling studies have identified miRNA-499 (miR499) in the heart;[6, 7] however, miR499 function is not fully elucidated. MiR499 is an evolutionarily conserved muscle-specific miRNA that is encoded within the intron of myosin heavy chain 7B *(Myh7B)* and is highly enriched in cardiac ventricular myocytes.[8, 9] miR499 has been demonstrated to be involved in the pathogenesis of valvular heart disease, ischemic heart disease, and heart failure.[10–12]

The cardiomyocytes are enriched with mitochondria that provide the ATP for the requisite continuous cardiac mechanical and electrical work.[13] Constant mitochondrial fusion and fission are necessary for the maintenance of organelle fidelity.[14–16] Mitochondrial dysfunction, as evidenced by abnormal mitochondrial fission and reduced ATP production, is a characteristic of the initiation of apoptosis in cardiomyocytes.[17] Calcineurin and dynamin related protein 1 (Drp1) have been shown to be involved in mitochondrial fission during cardiac apoptosis.[17, 18] Calcineurin contains a heterodimer of a 61-kD calmodulin-binding catalytic subunit, calcineurin A (CnA) contains a 19-kD Ca^2+^-binding regulatory subunit, and calcineurin B is a cytosolic serine and threonine phosphatase. Upon initiation of apoptosis, calcineurin dephosphorylates Drp1, leading to translocation of unphosphorylated Drp1 from the cytosol to the mitochondrial outer membrane resulting in mitochondrial fission and cellular apoptosis.[18, 19]

Overload via mechanical stretch induces an inflammatory response and can cause ventricular fibrosis and hypertrophy.[20, 21] Cyclic strain (repetitive stretching and relaxation) on cultured cells at rates comparable to dynamic stretch overload in vivo has been used to study the molecular mechanisms of genomic expression and signal transduction in cardiomyocytes, as well as vascular smooth muscle cells.[21, 22] Furthermore, mechanical stretch can also induce cellular apoptosis in cardiovascular cells.[22, 23] Liao et al. have demonstrated that mechanical stretch induces mitochondria-dependent apoptosis in neonatal rat cardiomyocytes.[23]

CnA is one of the target genes of miR499.[24] The expression of miR499 and its regulation of CnA in stretched cardiomyocytes remains to be fully elucidated. We currently hypothesize that CnA may be a target of miR499 in stretched cardiomyocytes in response to stress. Since volume overload is associated with heart failure and myocardial apoptosis,[25] the regulation of miR499 and CnA in ventricular volume overload was also investigated. The aims of the current study were to investigate whether the expression of miR499 in cardiomyocytes can be regulated with mechanical stretch and a rat model of volume-overload induced heart failure caused by aorta-caval shunt, and to evaluate the molecular mechanism for regulating miR499 on gene and protein expression.

## Methods

### Primary cardiomyocytes culture

Hearts from 2- to 3-day-old neonatal Wistar rats euthanized via cervical dislocation were dissociated using trypsin as previously described.[26, 27] The atrium and ventricle were separated prior to mincing. The minced tissues were then subjected to trypsin (0.125%) digestion in a balanced salt solution. The disaggregated cells were then collected following centrifugation (300 x g for 10 minutes). The cell pellet was resuspended in serum-containing medium (80% F10 nutrient mixture, 20% fetal bovine serum, 1% penicillin/streptomycin), plated into a Petri dish, and kept for 2.5 hours in a 5% CO_2_ atmosphere at 37°C to allow for cell attachment. The nonattached myocytes suspended in the medium were subsequently collected and plated at a density of 1.67 x 10^6^ cells/well on a six-well Flexcell I flexible membrane dishes coated with Collagen I (Flex I Culture Plates Collagen I; Flexcell International, Hillborough, NC, USA). After 2 days in culture, cardiomyocytes were transferred to serum-free medium (Ham’s F-12:DMEM Z 1:1) and maintained for another two days. Cultured cardiomyocytes were >90% pure (as evaluated via observation of contractile characteristics using a light microscope) and were stained using antidesmin antibody.

### In vitro cyclic stretch on cultured cardiac myocytes

The Flexcell FX-2000 strain unit (Flexcell International) consists of a vacuum unit linked to a valve controlled by a computer program. Cardiomyocytes cultured on the flexible membrane base were subjected to cyclic stretch through the application of sinusoidal negative pressure with a peak level of 15 kPa at a frequency of 1 Hz (60 cycles/min) for various periods of time.

### Rat model of aorta-caval (AV) shunt

An AV shunt was performed on adult Wistar rats to induce volume overload as previously described[28] (also see S1 File). After various weeks of AV shunt, rats were euthanized with an overdose of isoflurance. Tissue from the left ventricle was obtained for western blot analysis and histochemical staining. Hemodynamic monitoring of rats was performed using polyethylene catheters and a Grass model tachograph preamplifier. All animal procedures were performed in accordance with the Institutional Committee of Animal Care and Use (Protocol number #0990816001) and conformed to the Guide for the Care and Use of Laboratory Animals published by the US National Institutes of Health (NIH publication No. 86–23, revised 1996).

### Real-time Quantitative PCR

The primers used were as follows: CnA, 5’-d(CTGGTTCTTTGAGCGTGGAGGAGTT)-3’(forward) and 5’-d(CCATTCCCGTCTGTGTCGAAT)-3’ (reverse); and p53 5’-d(TATGGAAACTTCTTCCTCCAG)-3’(forward) and 5’-d(CCTTCTAACAACTCTGCAAC)-3’ (reversed); and internal control alpha-Tubulin 5’-d(GCACCTACCGCCAGCTCTT)-3’ (forward) and 5’-d(CAGCATCTTCCTTGCCTGTGA)-3’. Details of the procedures are further described in the S1 File.

### Quantitative analysis of microRNAs

Total RNA from cardiomyocytes was isolated using Trizol Reagent (InvitrogenTM, Life Technologies, Grand Island, NY, USA) according to the manufacturer’s instructions. TaqMan^®^ MiRNA realtime quantitative assays were used to quantitate miRNAs. Details of the procedures are further described in the S1 File.

### Protein isolation

Cardiomyocytes under mechanical stretch were harvested by scraping and then centrifuged (300 ug) for 10 minutes at 4°C. The pellet was resuspended and homogenized in a Reporter Lysis Buffer (Promega Corp., Madison, WI, USA), and centrifuged at 10,600 ug for 20 minutes. The protein content of the supernatant was quantified using Bio-Rad Protein Assay. Equal amounts of protein (30 μg) were loaded into a 10% SDS-polyacrylamide minigel, followed by electrophoresis.

### Calcineurin activity assay

Calcineurin cellular activity was measured using a calcineurin assay kit (Biomol, Plymouth Meeting, PA) according to manufacturer’s instructions and as previously described.[29] Briefly, calcineurin cellular activity was measured as the dephosphorylation rate of a synthetic phosphopeptide substrate. The detection of free-phosphopeptide substrate was determined using the Biomol Green reagent (Biomol, Plymouth Meeting, PA).

### Western blot analysis

A western blot was performed as previously described.[22] Briefly, rabbit polyclonal anti-CnA antibody,[30] anti-p-Drp1 (phosphorylated form), and anti-Drp1 (unphosphorylated form) antibody[31, 32] (1:200, Santa Cruz Biotechnology, Santa Cruz, USA) were used. Details of the procedures are further described in the S1 File.

### Construction and delivery of miR499, antagomir499, and mutant-miR499 expression vector into cultured cardiomyocytes and ventricular myocardium

A 85bp hsa-miR499 precursor construct as was generated as follows: (A) Genomic DNA was amplified with forward primer, CACGCCCTCTGCAGGC and reverse primer, CAGGACTCCCTCCCATGG. The 200bp amplified product was digested using EcoRI and BamHI restriction enzymes and ligated into pmR-ZsGreen1 plasmid vector (coexpression miR499 and green fluorescent protein, Clontech Laboratories, Mountain View, CA, USA). (B) MiR499 antagomir, and (C) mutant miR499 precursor construct was designed (Applied Biosystems) and ligated into the same plasmid vector as miR499. The constructed plasmid was transfected into cultured cardiomyoctyes or left ventricular myocardium using a low pressure-accelerated gene gun (Bioware Technologies, Taipei, Taiwan) per manufacturer instructions. Briefly, 2 mg of plasmid DNA was suspended in 5 ml of phosphate-buffered saline (PBS). Pushing the trigger of the low pressure gene gun released the DNA-containing solution, which was directly propelled by helium at a pressure of 15psi into the cultured cardiomyocytes or left ventricular myocardium. The distribution of treated cardiomyocytes or rats was visualized using a dissecting fluorescence microscope with high resolution CCD (Hama-Matsu Photonics, Japan). In the rat AV shunt model, the rat’s chest cavity was re-opened after 3 days, and the fluorescent image on left ventricular myocardium was detected indicative of a successful transfection. The transfection efficiency is depending on various cell type. Since primary cardiomyocytes was used in this study, the transfection efficiency of using this method was approximately 30% (S1 Fig).

### RNA interference

Cardiomyocytes were transfected using 800 ng small interfering RNA (siRNA) of CnA or p53 (Sigma, Singapore). Both CnA siRNA and p53 siRNA are target-specific 19 nucleotide siRNAs, according to a computer program provided by Dharmacon. The CnA siRNA and p53 siRNA targeted base sequences were as follows: sense, 5′-GAGUCUCUCAGUUCAGUGU and 5′-GAGAUGUUCCGAGAGCUGA-3′, respectively; and antisense, 5′-ACACUGAACUGAGAGACUC and 5′-UCAGCUCUCGGAACAUCUC-3, respectively. The CnA scramble siRNA and p53 scramble siRNA were used as a negative control, with the following base sequences: sense, 5′-GGCGUCUUUAUCGUGUACA and 5′-GGGGAUAGGUUACAUGCAC-3′, respectively; and antisense, 5′-UGUACACGAUAAAGACGCC and 5′-GUGCAUGUAACCUAUCCCC-3, respectively. After overnight incubation, cells were stretched and subjected to analysis by Western blot and quantitative analysis of microRNAs.

### Electrophoretic mobility shift assay (EMSA)

Nuclear protein concentrations from cultured cardiomyocytes were determined using a protein assay (Bio-Rad; Hercules, CA, USA). Consensus and control oligonucleotides (Santa Cruz Biotechnology, Santa Cruz, CA, USA) were labeled using polynucleotide kinase incorporation of [γ^32^-p]ATP. Oligonucleotide sequences of p53 were the consensus 5’-AGTATGGTCTAGCCTGGCCC-3’. The mutant oligonucleotides sequence was 5’-AGTATGGTACCACCTGGCCC-3’. After the p53 was radiolabelled, the nuclear extracts (4μg of protein in 2μL of nuclear extract) were mixed with 20pmol of the appropriate [γ^32^-p]ATP-labelled consensus or mutant oligonucleotides in a total volume 20μL for 30 min at room temperature. The samples were then resolved on a 4% polyacrylamide gel. Gels were dried and imaged using autoradiography. A control was performed in each case with mutant or cold oligonucleotides to compete with the labeled sequence.

### Promoter activity assay

A bp -941 to -442 rats miR499 promoter construct was generated as follows. Rat genomic DNA was amplified with forward primer 5’-ATAACGCGTAGGAATCTCCCCCTCT-3’ and reverse primer 5’-CCTAGATCTGTAAGTGATGGTCGTCC-3’. The amplified product was digested with Mlul and Bglll restriction enzymes and ligated into pGL3-basic luciferase plasmid vector (Mission Biotech, Taipei, Taiwan). The miR499 promoter contained p53 conserved sites (CTAG) at -694 to -690bp. Rat CnA promoter genomic DNA was amplified with forward primer 5’-TGACGCGTGTTTAATCCATCTCTGTTGG-3’ and reverse primer 5’-TGAGATCTCTTCTATCTGGCAAAGAAATTTTA-3’. The CnA promoter contained miR499 conserved sites (AAGCAGTCATGCAATGGCTTAA) at 908 to 929 bp. For the mutant, the p53 binding sites in miR499 promoter and miR499 binding site in CnA promoter were mutated using a mutagenesis kit (Stratagene, La Jolla, CA, USA). Site-specific mutations were confirmed using DNA sequencing. Plasmids were transfected into cardiomyocytes using a low pressure-accelerated gene gun per the manufacturer’s protocol (Bioware Technologies). Briefly, 2μg of plasmid DNA was suspended in 5μL of PBS and was delivered to the cultured cells at a helium pressure of 15psi. The transfective efficiency using this method was approximately 30%. The cell extracts were prepared using a dual luciferase reporter assay system (Promega, Madison, WI, USA) and measured for dual luciferase activity with a luminometer (Turner Designs, Sunnyvale, CA, USA). Renilla luciferase activity was normalized to firefly luciferase activity.

### Cytotoxicity studies

Cell viability after the application of cyclic stretch was monitored using a trypan blue staining procedure and the 3-(4,5-cimethylthiazol-2-yl)-2,5-diphenyl tetrazolium bromide (MTT) assay to detect for stretch-induced cell injury. Cytotoxicity studies were performed as previously described.[22]

### Using flow cytometric analysis for apoptotic quantification

Apoptotic cells were quantified as the percentage of cells with hypodiploid DNA (sub-G1). Cardiomyocytes were fixed with 70% ethanol and treated with RNase. Nuclei were then stained with propidium iodide (Molecular Probes, Eugene, OR, USA) and fluorescein isothiocyanate–annexin V. Cardiomyocytes that were negative for both annexin V and propidum iodide were considered to be alive. Cells that were positive for annexin V and negative for propidium iodide were considered to be undergoing apoptosis. Cells that were positive for both annexin V and propidium iodide were considered to be in the end stage of apoptosis, called second apoptosis. DNA content was measured using a FACSCalibur flow cytometer and Cell Quest software (Becton Dickinson, Franklin Lakes, NJ, USA). Ten thousand cells were counted in all assays.

### Assessment of cellular ATP levels, protease activity, and caspase-3 activity assay

Cellular ATP levels and protease activity were measured after a 90-minute incubation using the Mitochrondrial ToxGlo assay (Promega, USA) as per manufacturer’s instructions. These 2 sets of data were combined to represent mitochrondrial dysfunction-related cytotoxic mechanisms. Apoptosis was also determined via caspase-3 activity using the caspase-3 colorimetric activity assay kit (Millipore, Temecula, CA). The stretched cardiomyocytes were resuspended in chilled 1X Cell Lysis buffer and then centrifuged for 5 mins (10000 x g). The supernatant was then transferred to a fresh tube, adding 5X assay buffer and caspase-3 substrate. After incubating for 2 hours at 37°C, the sample was evaluated at 405 nm in a microtiter plate reader.

### Terminal deoxynucleotidyl transferase-mediated dUTP nick-end labeling (TUNEL) assay

DNA fragmentation was determined via TUNEL assay using the ApopTag peroxidase in situ apoptosis detection kit (Chemicon International, Temecula, CA, USA). The methodology for the TUNEL assay is further described in detail in the S1 File.

### Immunohistochemical analysis and In situ hybridization assay

The methodologies for the immunohistochemical analysis and in situ hybridization assay in the AV shunt rat model are further described in detail in the S1 File.

### Statistical analysis

All results are expressed as means ± SEM. Statistical significance was evaluated using analysis of variance (ANOVA; GraphPad Software Inc., San Diego, CA, USA). Dunnett’s test was used to compare multiple groups with a single control group. The Turkey–Kramer comparison was used for pair-wise comparisons between multiple groups following the ANOVA. A value of P < 0.05 was considered as significant.

## Results

### Mechanical stretch inhibits miR499, but increases Calcineurin A (CnA) mRNA, CnA, and unphosphorylated Drp1 protein expression in cardiomyocytes

**Fig 1:**
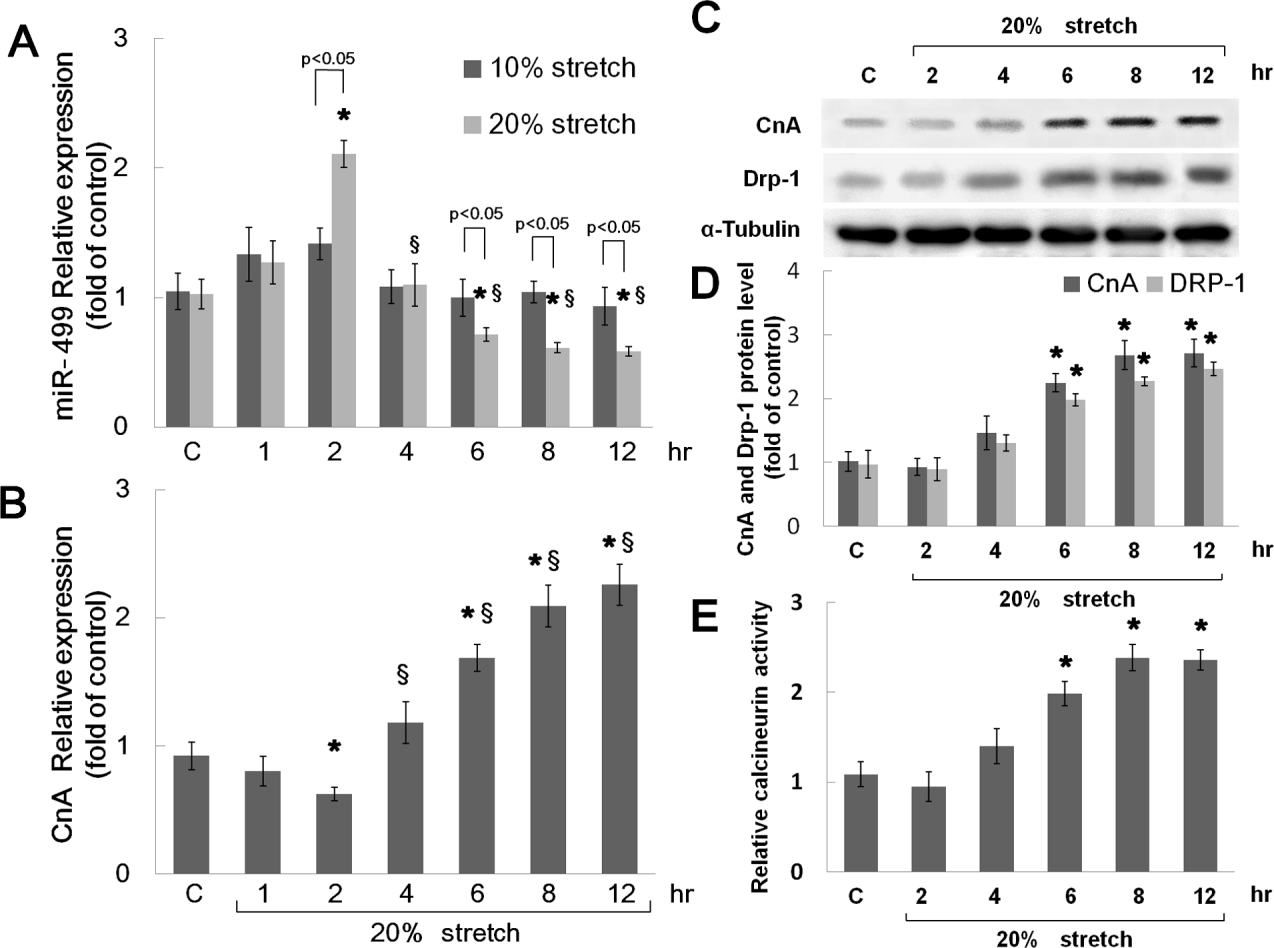
(A) Representative expression of miR499 in cardiomyocytes subjected to 10% or 20% mechanical stretch for 0–12 hours. (B) Fold increases in mRNA of CnA as a result of mechanical stretch for 0–12 hours. All fold changes between samples were determined using the comparative C_T_ method (n = 5 per group). (C) Representative Western blot for CnA and dephosphorylated Drp1 protein in cardiomyocytes subjected to 20% stretch for 0–12 hours. (D) Quantitative analysis of CnA and dephosphorylated Drp1 protein levels. The values from stretched cardiomyocytes have been normalized to control cell values (n = 4 per group). (E) Calcineurin cellular activity in cardiomyocytes subjected to 20% stretch for 0–12 hours. * p < 0.001 vs. control, § p < 0.001 vs. 20% stretch for 2 hours.

To investigate the effect of mechanical stretch on miR499 expression in cardiomyocytes, TaqMan MicroRNA real-time quantitative PCR (Applied Biosystems) was used to measure miR499. When cardiomyocytes were stretched to 10% of elongation, miR499 levels were similar to those of control cells (no stretch). Mechanical stretch of 20% for 2 hours resulted in significant miR499 expression compared with control cells, however stretch for 4 to 12 hours significantly down regulated miR499 expression (Fig 1A) compared with the control and 2 hour stretch condition (Fig 1A). CnA mRNA expression was induced by mechanical stretch of 20% for 6 to 12 hours compared with control cells (Fig 1B). Mechanical stretch for 6 to 12 hours significantly increased CnA and unphosphorylated Drp1 protein expression in cardiomyocytes (Fig 1C and 1D). The relative activity of Calcineurin was significantly increased with mechanical stretch for 6 to 12 hours (Fig 1E).

### Mechanical stretch increases expression of CnA, unphosphorylated Drp1, and Calcineurin cellular activity via miR499

**Fig 2:**
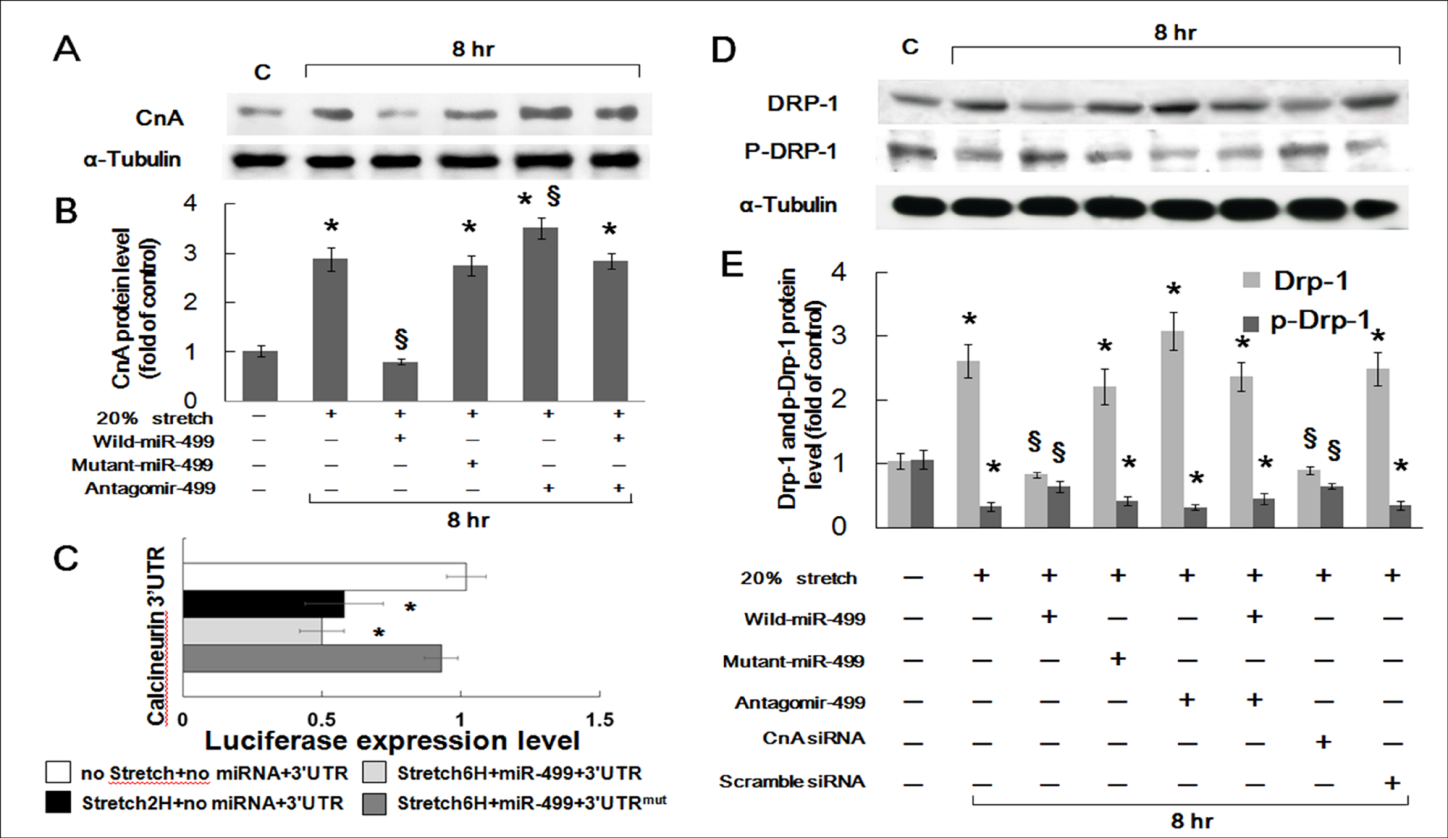
(A and B) Representative Western blot and quantitative analysis for the expression of CnA in cardiomyocytes subjected to 20% stretch for 8 hours with the overexpression of miR499, mutant miR499, or antagomir499 (n = 4 per group). (C) The miR-499 binding site in the 3′ UTR of CnA mRNA specifically mediated miR-499-dependent repression in luciferase assays. (D) Representative Western blot for dephosphorylated (Drp-1) and phosphorylated Drp1 (p-Drp1) protein in cardiomyocytes subjected to stretch at 20% elongation for 8 hours with the overexpression of miR499, mutant miR499, antagomir499, CnA siRNA, and scramble siRNA. (B) Quantitative analysis of Drp1 and p-Drp1 protein levels in stretched cardiomyocytes with overexpression of miR499, mutant miR499, antagomir499, CnA siRNA, and scramble siRNA. * p < 0.001 vs. control; § p < 0.001 vs. stretch alone.

Overexpression of miR499 significantly inhibited CnA expression (Fig 2A and 2B) and Calcineurin cellular activity (S2 Fig) compared with stretch only; whereas, the addition of mutant miR499 had no effect on CnA expression and calcineurin activity induced by mechanical stretch for 8 hours. Antagomir499 alone did not affect the expression of CnA and calcineurin activity induced by stretch; however, antagomir499 attenuated the inhibitory effect of miR499 overexpression on CnA and calcineurin cellular activity. The miR-499 binding site in the 3′ UTR of CnA mRNA specifically mediated miR-499-dependent repression in luciferase assays (Fig 2C).

MiR499 regulation of Drp1 accumulation in cardiomyocytes through its effects on CnA expression was also examined. The expression of unphosphorylated (Drp1) and phosphorylated (p-Drp1) were induced by mechanical stretch of 20% for 8 hours (Fig 2D and 2E). Mechanical stretch significantly increased the accumulation of Drp1, but decreased p-Drp1 levels in cardiomyocytes. MiR499 overexpression attenuated Drp1 accumulation in stretched cardiomyocytes. Mutant miR499 had no effect on the expression of Drp1 induced by stretch. The addition of antagomir499 alone increased expression of Drp1 more than stretch alone; whereas, antagomir499 attenuated the effect of miR499 overexpression on Drp1 inhibition. CnA siRNA inhibited the expression of Drp1, whereas scramble siRNA had no effect on Drp1 expression. These results suggest that miR499 attenuated Drp1 accumulation via CnA in stretched cardiomyocytes.

### Inhibition of p53 increases miR499 expression

**Fig 3:**
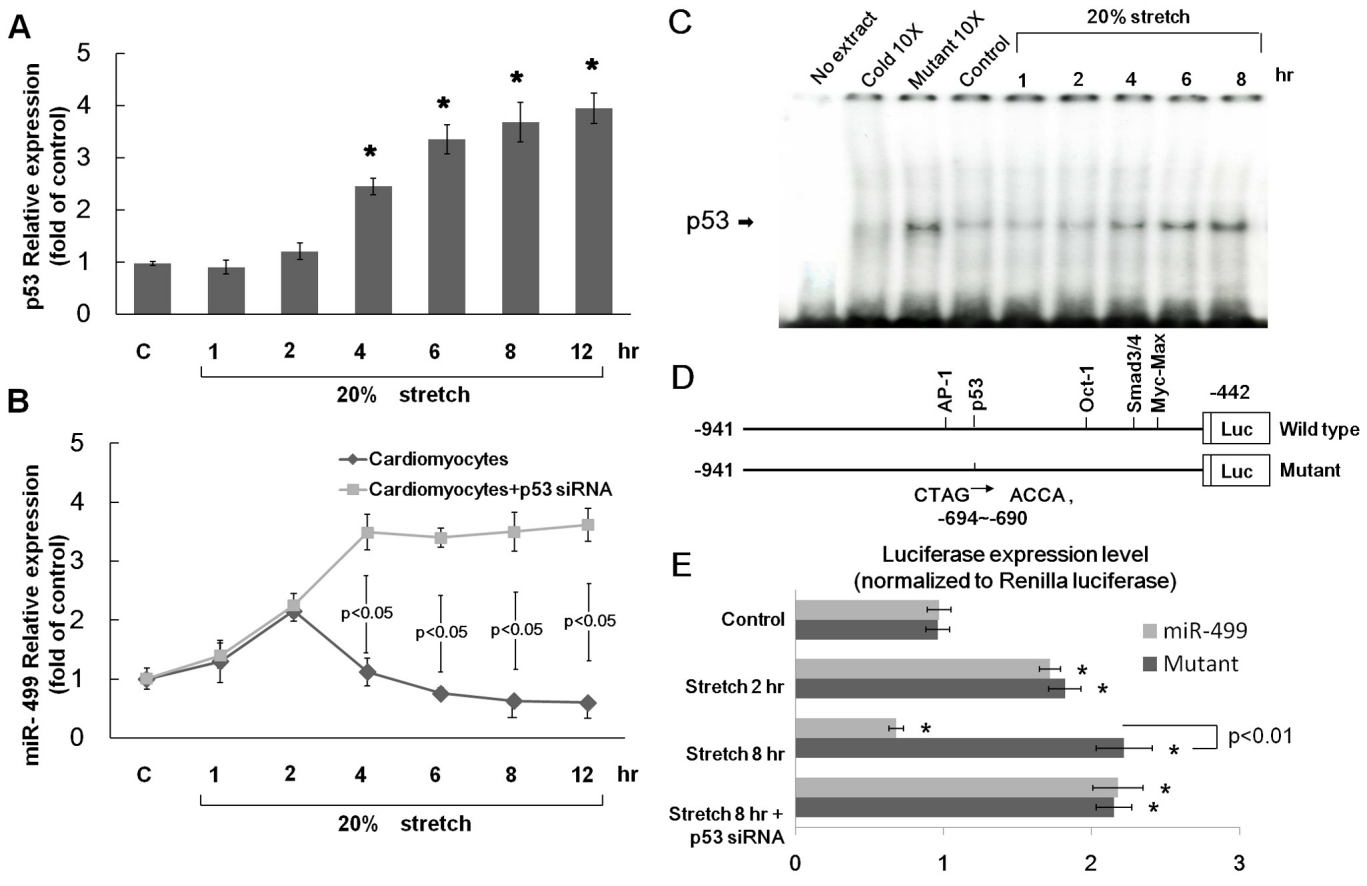
(A) Fold increases in p53 mRNA with mechanical stretch for 0–12 hours. All fold changes between samples were determined using the comparative C_T_ method (n = 5 per group). * p < 0.001 vs. control. (B) The expression of miR499 with 20% stretch for 0–12 hours with and without the addition of p53 siRNA. (C) Representative EMSA showing protein binding to the p53 oligonucleotide in cardiomyocyte nuclear extracts subjected to 20% stretch for 1–8 hours. (D) Constructs of the wild type and mutant miR499 promoter. The mutant miR499 promoter has a mutation of p53-binding sites in the miR499 promoter region as indicated. (E) Quantitative analysis of miR499 promoter activity. Cultured cardiomyocytes were transiently transfected with pmiR499-Luc. Luciferase activity in cell lysates was measured and normalized by renilla activity using a dual-luciferase assay system (n = 5 per group). * p < 0.001 vs. control.

The involvement of p53 in the regulation of miR499 expression in mechanical stretch was examined. p53 expression was induced by mechanical stretch of 20% for 4 to 12 hours (Fig 3A). The expression of miR499 in cardiomyocytes with and without the addition of p53 siRNA resulting from mechanical stretch is shown in Fig 3B. The addition of p53 siRNA significantly reversed the expression of miR499 with mechanical stress for 4 to 12 hours compared with cardiomyocytes without p53 siRNA. These data suggest that p53 can negatively regulate miR499 expression.

### Mechanical stretch increases p53 DNA binding activity and suppresses miR499 promoter activity

Mechanical stretch of cardiomyocytes for 2 to 8 hours increased p53 DNA binding activity (Fig 3C). To study whether the repression of miR499 by mechanical stretch is regulated at the transcriptional level, we cloned the promoter region of rat miR499 and constructed a luciferase reporter plasmid (pGL3-Luc). The miR499 promoter construct contained AP-1-, p53-, Oct-1, Smad3/4-, and Myc-Max-binding sites (Fig 3D). The AP-1, Oct1, Smad3/4-, and Myc-Max- DNA binding activity did not change significantly after mechanical stretch (S3 Fig). The mutant miR499 promoter has a mutation of p53-binding sites. A transient transfection of this reporter gene into cardiomyocytes revealed that mechanical stretch at 2 hours induced miR499 promoter activation but stretch at 8 hours suppressed its activation (Fig 3E). This result indicated that miR499 was suppressed at the transcriptional level when cells were exposed to mechanical stretch. When the p53 binding sites were mutated, the inhibition of miR499 promoter activity by stretch at 8 hours was abolished. Moreover, the addition of p53 siRNA reversed the inhibition of miR499 promoter activity under stretch at 8 hours. These results suggest that the p53 binding site in the miR499 promoter is essential for the transcriptional regulation induced by mechanical stretch and that mechanical stretch regulates the miR499 promoter via the p53 pathway.

### Mechanical stretch-induced apoptosis is mediated by miR499 in cardiomyocytes

**Fig 4:**
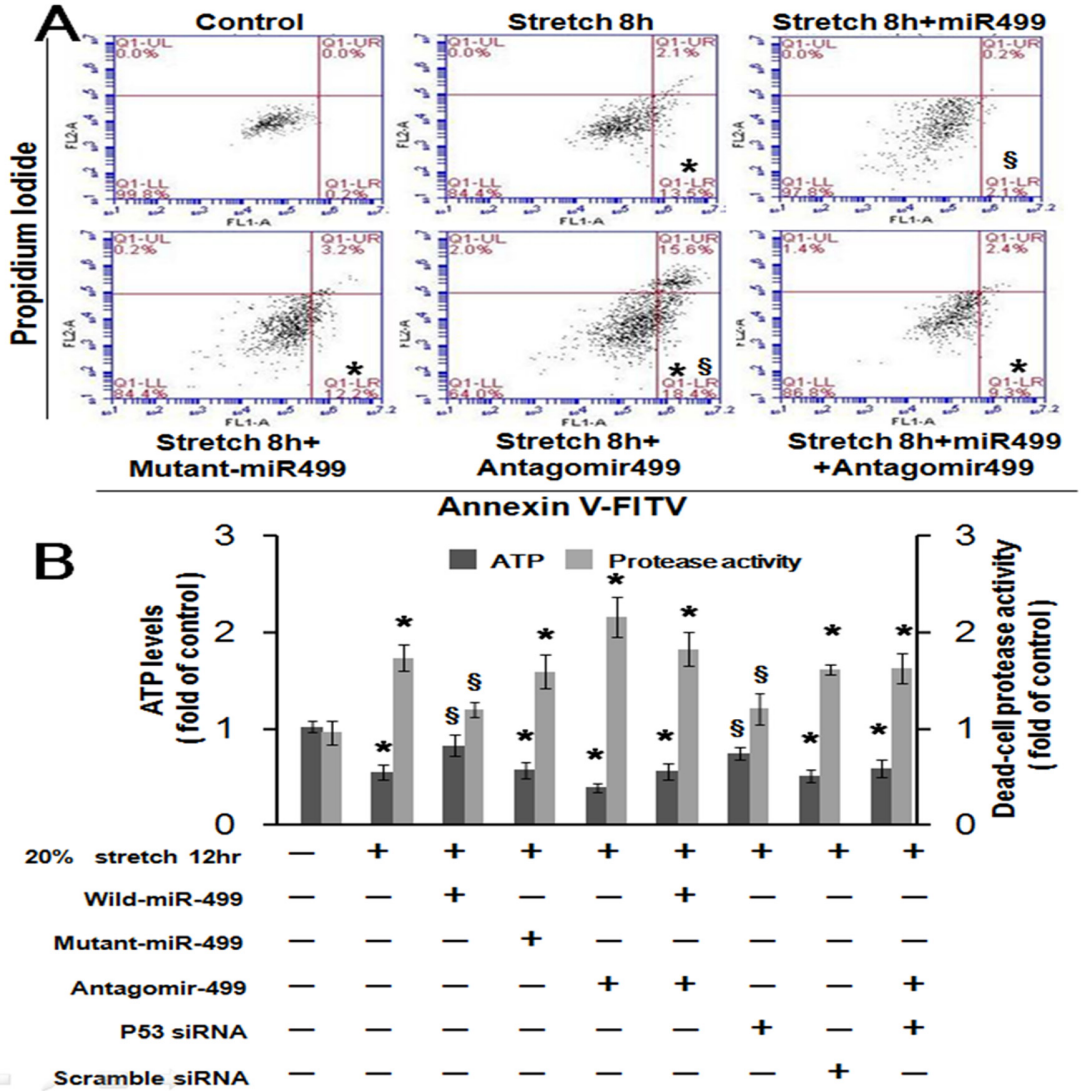
(A) Cardiomyocytes were subjected to mechanical stretch for 8 hours with miR499, mutant miR499, or antagomir499 overexpression. Quantification of the apoptotic fraction was performed using FACScan (n = 4). Cells that stain negative for both annexin V and propidium iodide are alive. Cells that stain positive for annexin V and negative for propidium iodide are undergoing apoptosis. Cells that stain positive for both annexin V and propidium iodide are in the end stage of apoptosis, or second apoptosis. * p < 0.001 vs. stretch. (B) Quantitative analysis of cellular ATP activity and protease activity in the stretched cardiomyocytes with overexpression of miR499, mutant miR499, antagomir499, P53 siRNA, and scramble siRNA. * p < 0.001 vs. control.

Apoptosis was assessed using propidium iodide-annexin V double staining and FACS analysis. The percentage of cells stained with annexin V was elevated following stretch for 8 hours (Fig 4A). The observed annexin V increases were significantly reversed by overexpression of miR499. Mutant miR499 alone had no effect on apoptosis. Antagomir499 enhanced cardiomyocyte apoptosis that had been suppressed by the overexpression of miR499. These results indicate that miR499 plays an essential role in cardiomyocyte apoptosis when exposed to mechanical stretch. Similarly, caspase 3 activity was induced by mechanical stretch (S4 Fig). The addition of miR499 to cardiomyocytes decreased caspase 3 activity. Conversely, the addition of antagomir499 enhanced the activity of caspase 3 under mechanical stretch. In addition, antagomir499 abolished the suppression of caspase 3 activity under stretch by miR499 overexpression.

### Hemodynamic and echocardiographic change after AV shunting

The influence of miR499 on the expression of CnA in vivo was examined. AV shunt was performed in adult Sprague-Dawley rats to induce volume overload. Heart weight and heart weight/body weight ratio significantly increased for 14 and 28 days after AV shunt (S1 Table). The heart rate and mean arterial blood pressure did not change significantly. LV end-diastolic (LVEDD) and end-systolic dimensions (LVESD) significantly increased after AV shunt at 28 days, while inter-ventricular septum thickness and left ventricular posterior wall thickness did not significantly change, indicating the volume overload induced by AV shunt.

### AV shunt inhibits myocardial miR499, but increases calcineurin A (CnA) protein expression

**Fig 5:**
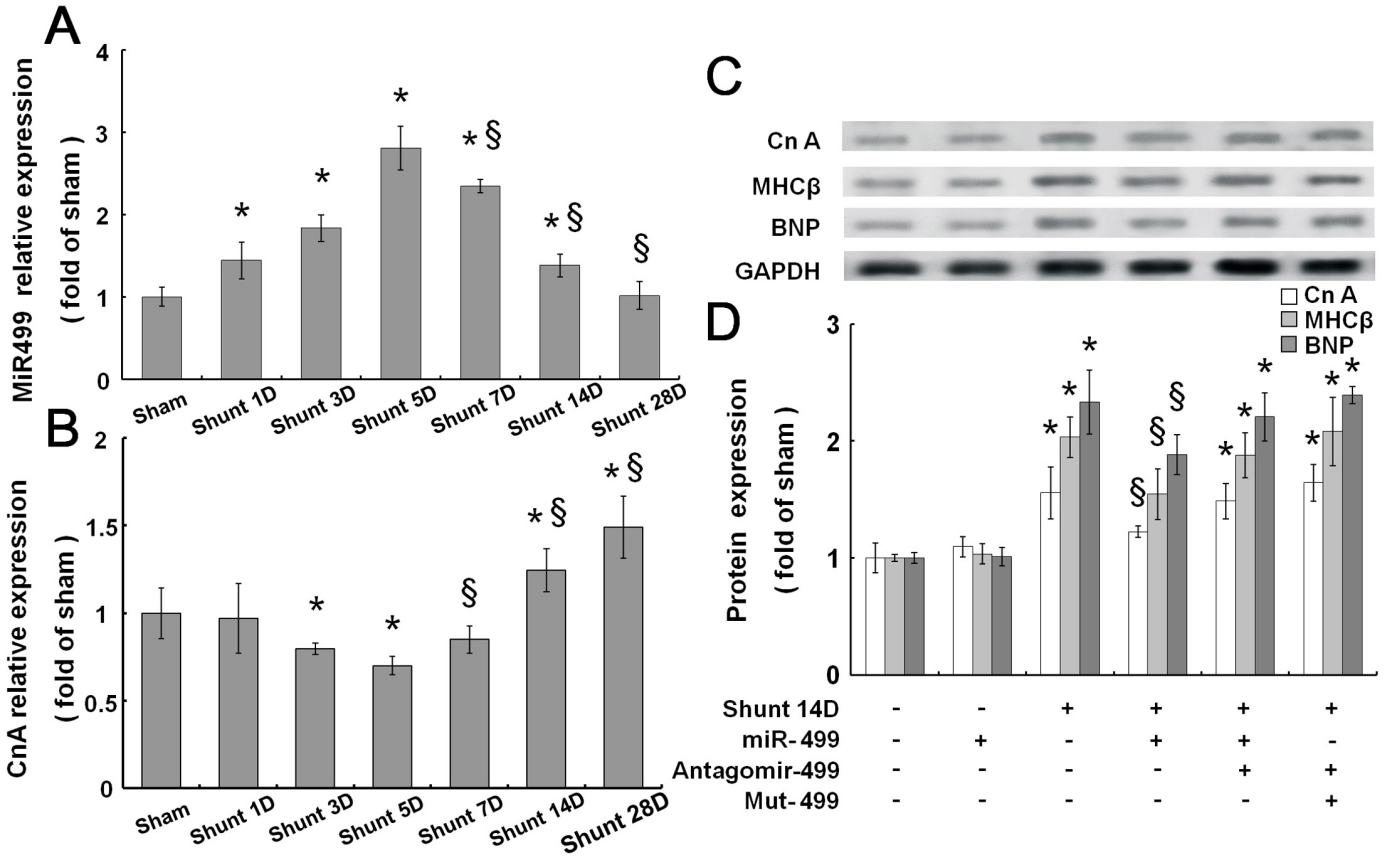
Representative expression of miR499 (A) and CnA protein (B) of left ventricular myocardium in aorta-caval (AV) shunt rats for 1 to 28 days. (C) Western blot for CnA, MHC-ß, and BNP protein expression from left ventricular myocardium after 14 days of shunting with miR499, mutant miR499, and antagomir499 overexpression,. (D) Quantitative analysis of CnA, MHC-ß, and BNP protein levels of the left ventricular myocardium in AV shunt rats. * p < 0.001 vs. control.

AV shunt significantly increased myocardial miR499 expression from 1 day to 5 days after shunting, but later decreased significantly from 7 days to 28 days compared to AV shunt at 5 days (Fig 5A). These findings suggest that myocardial miR499 levels are downregulated in volume overloaded heart. AV shunt significantly increased myocardial CnA protein expression from 14 days to 28 days (Fig 5B).

### Mir499 mediates the myocardial Calcineurin A (CnA) expression

To investigate the effect of miR499 on myocardial CnA expression, overexpression of miR499, antagomir-499, and mutant type miR499 (mut-499) in the left ventricle was performed. AV shunt at 14 days significantly increased myocardial CnA protein expression (Fig 5C and 5D). The cardiac hypertrophic markers such as MHCß and BNP were also significantly induced by AV shunt at 14 days. Overexpression of miR499 significantly decreased myocardial CnA, MHCß, and BNP expression induced by AV shunt. Overexpression of antagomir-499 or mutant miR499 (mut-499) did not have an effect on myocardial CnA protein expression.

**Fig 6:**
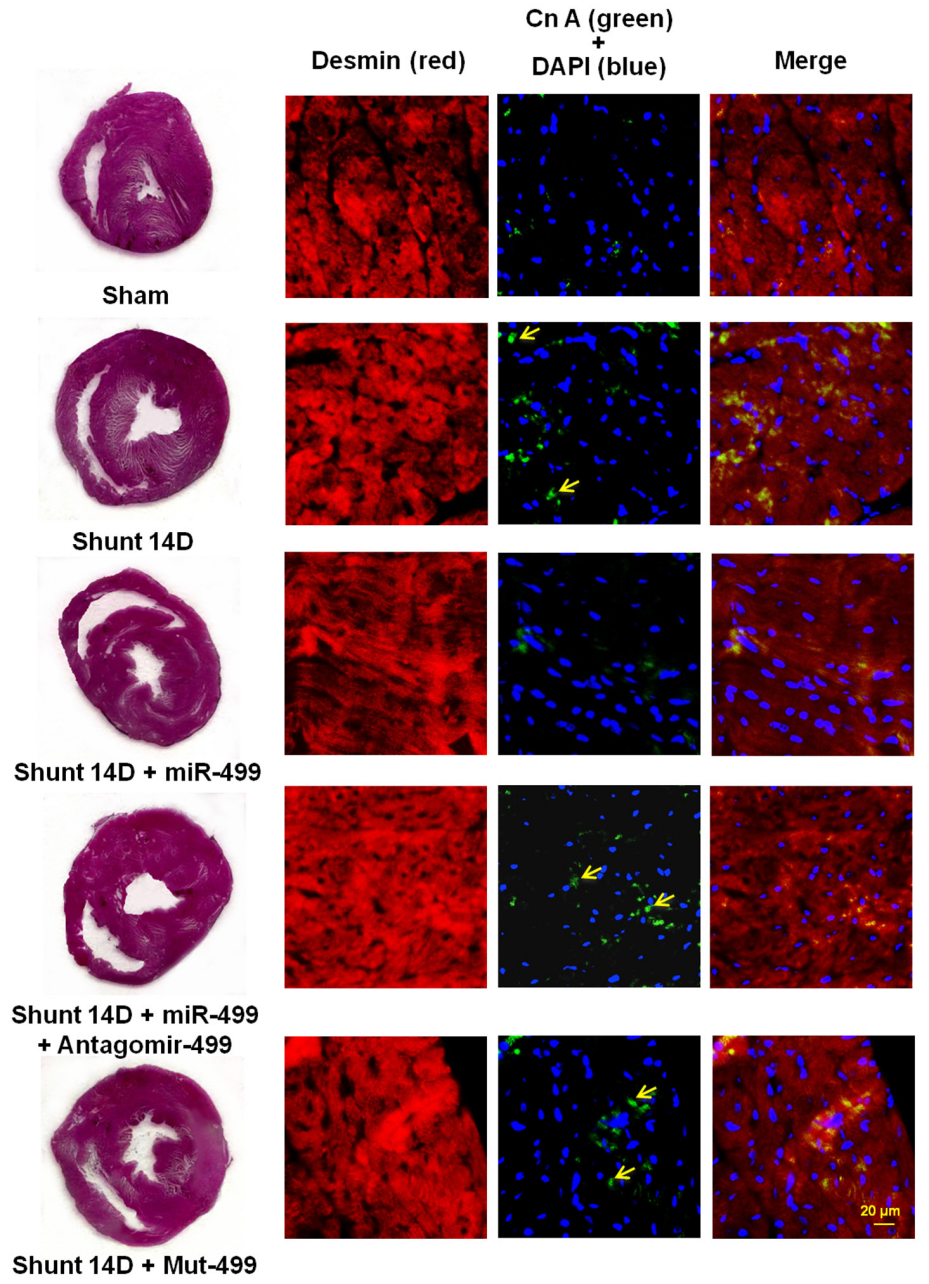
Immunohistochemical staining of left ventricular myocardium after induction of aorta-caval (AV) shunt with or without overexpression of mir499 or antagomir 499 treatments. There are significantly increased immunoreactive signals for CnA after AV shunt for 14 days. Overexpression of miR499 significantly decreased the immunoreactive signal induced by AV shunt. Rare CnA signals were seen in the sham group.

At 14 days after AV shunt, the presence of miR499 in cardiomyocyte cytoplasm was confirmed using in situ hybridization (S5 Fig). Immunohistochemical staining also showed increased myocardial CnA expression after AV shunt at 14 days (Fig 6). Overexpression of miR499 decreased the expression of CnA, which could be reversed by adding antagomir 499. Mutant miR499 did not change myocardial CnA after AV shunt. Myocardial CnA was not stained in the control sham group.

### AV shunt increased myocardial cellular apoptosis via miR499

**Fig 7:**
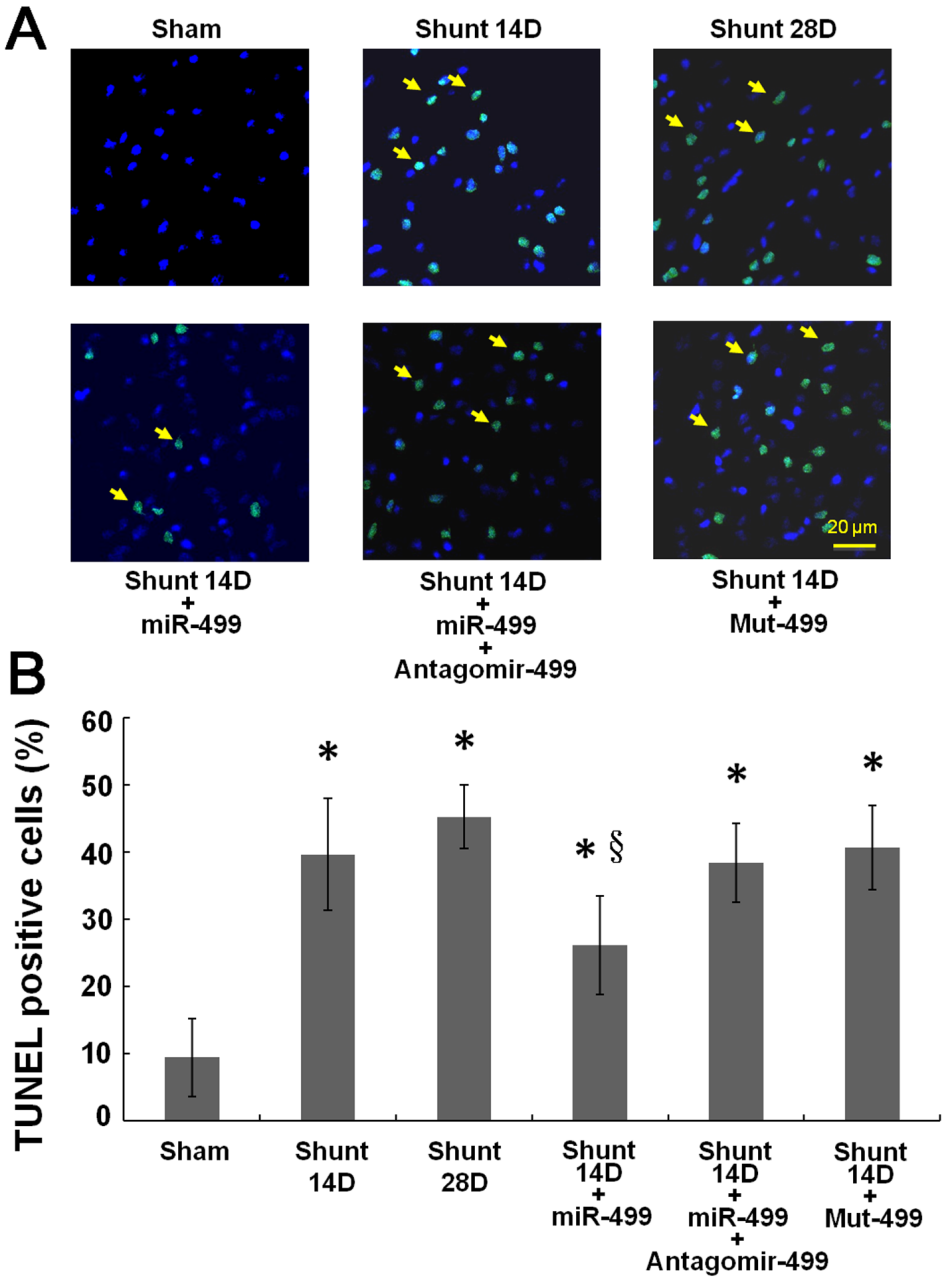
(A) Representative microscopy images of myocardium after volume overload heart, addition of miR499 overexpression, mutant miR499 or Antagomir499 before being stretched. TUNEL staining is indicative of cell death. Arrows indicate TUNEL-positive cardiomyocytes. (B) The quantitative analysis of TUNEL assay.

A significant increase in TUNEL positive nuclei was present in AV shunt hearts (Fig 7 and S6 Fig). Increases in TUNEL positive nuclei of cardiomyocytes induced by AV shunt were significantly reversed by overexpression of miR499. Adding antagomir 499 attenuated the effect of miR499, while mutant miR499 did not have an effect on myocardial cellular apoptosis after AV shunt. These findings demonstrate that miR499 mediates myocardial cellular apoptosis induced by AV shunt.

The heart weight, heart weight/body weight ratio, LVEDD and LVESD were significantly improved after overexpression of miR499. The fraction shortening (FS) of LV was also improved but statically insignificant. Adding antagomir 499 attenuated the effect of miR499, while mutant miR499 have no effect on heart size after AV shunt. (S1 Table)

## Discussion

We currently report the following major findings: (i) mechanical stretch, as well as AV shunt, down regulates miR499 in cardiomyocytes; (ii) mechanical stretch of cardiomyocytes induces the expression of CnA at the protein and mRNA levels, and cellular activity of calcineurin; (iii) expression of CnA and calcineurin activity are suppressed by miR499 during stretch; (iv) when exposed to mechanical stretch, dephosphorylated Drp1 expression is suppressed by overexpression of miR499 and CnA siRNA; (v) mechanical stretch induces apoptosis of cardiomyocytes via an miR499-controlled apoptotic pathway involving CnA and Drp1; (vi) suppression of p53 reverses the transcriptional downregulation of miR499 by mechanical stretch; (vii) CnA and myocardial cellular apoptosis were induced in heart failure induced by volume overload; and (viii) overexpression of miR499 suppressed CnA and attenuated myocardial cellular apoptosis after AV shunt.

Mechanical stretch triggers cardiomyocyte remodeling characterized by loss of contractile tissue, hypertrophy, and increased fibrotic tissue.[20] Several recent studies suggest that mechanical stretch simultaneously causes cardiomyocytes apoptosis and cardiac fibroblast proliferation, resulting decreased contraction ability and increased fibrous tissue in myocardium.[20, 33] Cardiomyocyte apoptosis and cardiac fibrosis are critical events in cardiac remodeling and the possible transition to heart failure. Previous studies have also described significant mitochondrial changes during heart failure. Abnormal mitochondrial morphology, reduced mitochondrial volume density, and altered levels of most electron transport chain proteins have been observed in rat models of systolic dysfunction induced by aortic constriction, rapid pacing, and myocardial infarction.[34–36] Abnormal mitochondrial morphology or function is implicated in the initiation of apoptosis.[18, 37] Several miRNAs have been reported to be involved in cardiomyocyte apoptosis, such as miRNA 1, 9b, 34a, 101, 122, 200a, 320, and 449, which are pro-apoptotic; whereas, miRNA 21, 30, 125b, 133, and 206 are anti-apoptotic.[38] The present study is the first to demonstrate that miR499 is actively involved in the inhibition of apoptosis induced by mechanical stretch and volume overload heart failure.

We currently demonstrate significant miR499 down regulation with mechanical stretch and volume overload. This down-regulation results in increased CnA and expression of dephosphorylated Drp1due to mitochondrial fission and cellular apoptosis. Overexpression of miR499 reduced CnA and dephosphorylated Drp1 protein levels, whereas application of antagomir-499 caused a robust increase in CnA and consequent dephosphorylated Drp1 protein, indicating a relief of tonic repression of CnA and dephosphorylated Drp1 by miR499. Taken together, these results suggest that miR499 plays an essential role in the synthesis of CnA and dephosphorylated Drp1 when placed under mechanical stretch and may provide important new information on the role of miR499 in CnA-Drp1–mediated apoptosis.

In the present study, while miR499 was down regulated 4 to 12 hours after mechanical stretch, CnA and dephosphorylated Drp1 protein levels were consistently increased after 6 to 12 hours of stretch. These findings are consistent with previous observations that CnA expression and activity of calcineurin increases under physiological stressful conditions, including heart failure[39] and hypoxia[40], and that inhibition of CnA can attenuate myocardial infarction.[41] The current study has demonstrated that the addition of CnA siRNA inhibited the expression of dephosphorylated Drp1. CnA provokes apoptosis by dephosphorylating apoptotic factors.[17] Several reports have proposed the CnA-mediated dephosphorylation of Drp1 regulates Drp1 function, including the promotion of mitochondrial fragmentation,[42] and fission.[37] Control of the CnA-Drp1 apoptotic pathway by miR499 may play a role in blocking the effects of pathological insults to the heart.

MiR499 expression may be down regulated by mechanical stretch. The level of p53 is increased after mechanical stretch and is a modulator of cardiomyocyte apoptosis after stretch.[23, 43] These findings led us to examine whether p53 is involved in the regulation of miR499 expression with mechanical stretch. Consistent with previous studies, we observed significantly increased levels of p53 following 4 to 12 hours of mechanical stretch. [23, 43] Correspondingly, the addition of p53 siRNA reversed the downregulation of miR499 during mechanical stretch.

The promoter activity analysis verified the regulation of miR499 expression at the transcriptional level when cardiomyocytes were placed under mechanical stretch. Mechanical stretch increased the binding activity of p53 to DNA and the removal of the p53-binding site in the miR499 promoter area abolished the suppression effect of p53 on miR499 promoter activity. These finding suggest the decreased transcriptional activity of the miR499 promoter due to mechanical stretch is depended on p53. Therefore, it is likely that enhanced p53 activity associated with mechanical stretch down-regulates miR499 transcription, which results in relief of repression of CnA, and thereby an increase in dephosphorylated Drp1, leading to mitochondrial fission and cardiomyocytes apoptosis. In addition, the current report is supported by Liao et al. who demonstrated that p53 is upregulated at relatively late time after mechanical stretch.[23] The regulatory effects of p53 on miR499 may occur as late as 4 to 12 hours after mechanical stretch.

Previous study by Wang J et al had demonstrated that miR499 protects cardiomyocytes from H_2_O_2_-induced injury via its effects on Pdcd4 and Pacs2.[44] In the present study, miR499-controlled apoptotic pathway involving CnA and Drp1 in cardiomyocytes under mechanical stretch may be regulated by p53. Whether miR499 has other potential targets in protecting cardiomyocytes from apoptosis remains to be determined. However, these findings suggested miR499 may protect cardiomyocytes from apoptosis induced by different stress, such as reactive oxygen species (ROS) and mechanical stretch.

## Conclusion

The current study is the first to demonstrate a link between p53, miR499, CnA, and Drp1 and cardiomyocyte apoptosis when placed under mechanical stretch. CnA is a direct target of miR499 and miR499 inhibits cardiomyocyte apoptosis through the suppression of CnA-mediated Drp1, thereby decreasing Drp1-mediated activation of mitochondrial fission. We also observed the down regulation of miR499 expression by p53 transcription when placed under mechanical stretch. Overexpression of miR499 attenuated cardiomyocyte apoptosis in heart failure induced by volume overload. Modulation of miR499 levels could provide a therapeutic approach for treating heart failure.

## Supporting Information

S1 Fig Using transfection reagent for delivery of miR499 expression vector into cultured cardiomyocytes, the transfection efficiency is around 30–40%. The green spot is miR499 in situ image.
(TIF)

S2 Fig Representative EMSA showing protein binding to the AP1, Oct1, Smad3/4 and Myc-Max oligonucleotide in cardiomyocyte nuclear extracts subjected to 20% stretch for 1–8 hours. (TIF)

S3 Fig Quantitative analysis of Caspase 3 activity in the stretched cardiomyocytes with overexpression of miR499, mutant miR499, and antagomir499. * p < 0.001 vs. control. § p < 0.001 vs. stretch alone.
(TIF)

S4 Fig Hemodynamic and echocardiographic parameters of the failing heart induced by aorta-caval shunt. (TIF)

S5 Fig In situ hybridization assay detects the presence of miR499 in the cardiac myocytes. Representative microscopic images showing the presence of miR499 (green color) in the cytoplasm of cardiac myocytes from left ventricular myocardium in AV shunt rats. The sham groups or scrambled probe did not detect the presence of miR499.
(TIF)

S6 Fig Representative microscopy images (in higher resolution) of myocardium in sham group. TUNEL staining is indicative of cell death.
(TIF)

S1 File Supplement Methods. (DOC)

S1 Table Calcineurin cellular activity in cardiomyocytes with 20% stretch for 8 hours with the overexpression of miR499, mutant miR499, or antagomir499 (n = 5 per group). (DOC)
