# Correction: Mechanical Stretch Inhibits MicroRNA499 via p53 to Regulate Calcineurin-A Expression in Rat Cardiomyocytes

**DOI:** 10.1371/journal.pone.0158257

**Published:** 2016-06-23

**Authors:** Su-Kiat Chual, Bao-Wei Wang, Huey-Ming Lo, Yuh-Feng Lin, Chiung-Zuan Chiu, Li-Ming Lien, Kou-Gi Shyul

There is an error in the byline. Prof. Lin Yun-Feng should be included as the fourth author. The correct affiliation for Prof. Lin Yun-Feng should be #1: Graduate Institute of Clinical Medicine, College of Medicine, Taipei Medical University, Taipei, Taiwan. The contributions to the manuscript are as follows: Conception and the design of the work, acquisition of data, or analysis or interpretation of data; final approval of the version to be published.
