# Supplementary figures and images for: Mechanical Stretch Inhibits MicroRNA499 via p53 to Regulate Calcineurin-A Expression in Rat Cardiomyocytes
Source: PLoS One. 2016 Feb 9;11(2):e0148683. doi: 10.1371/journal.pone.0148683 (PMC4747570; doi:10.1371/journal.pone.0148683)

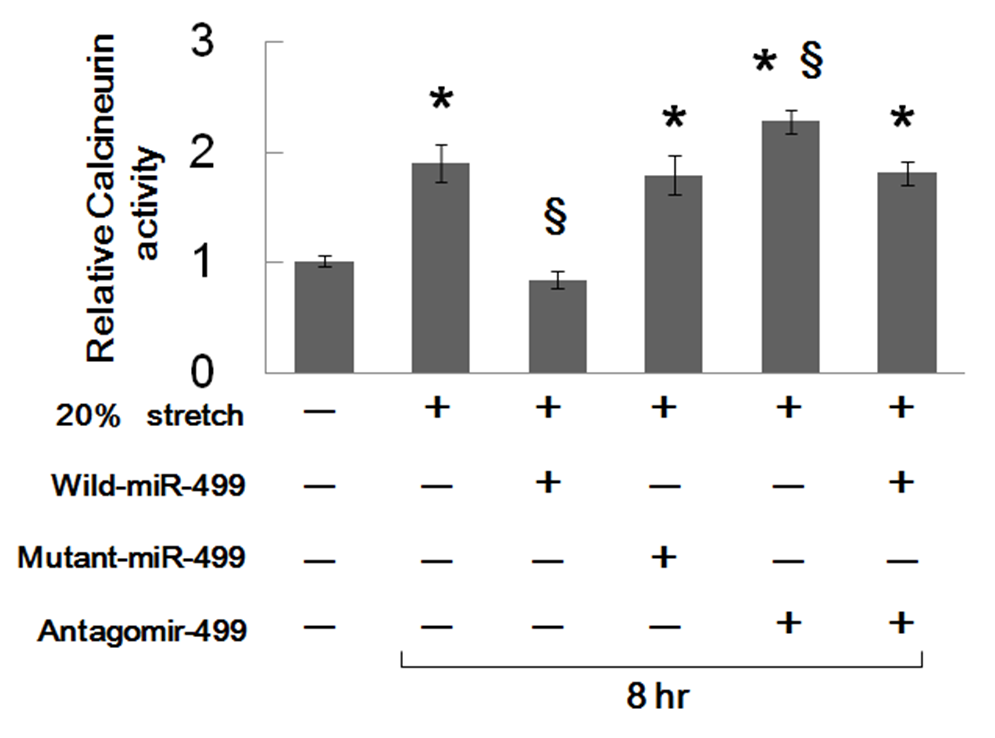

Supplement: S1 Fig — The green spot is miR499 in situ image. (TIF) [file pone.0148683.s001.tif]

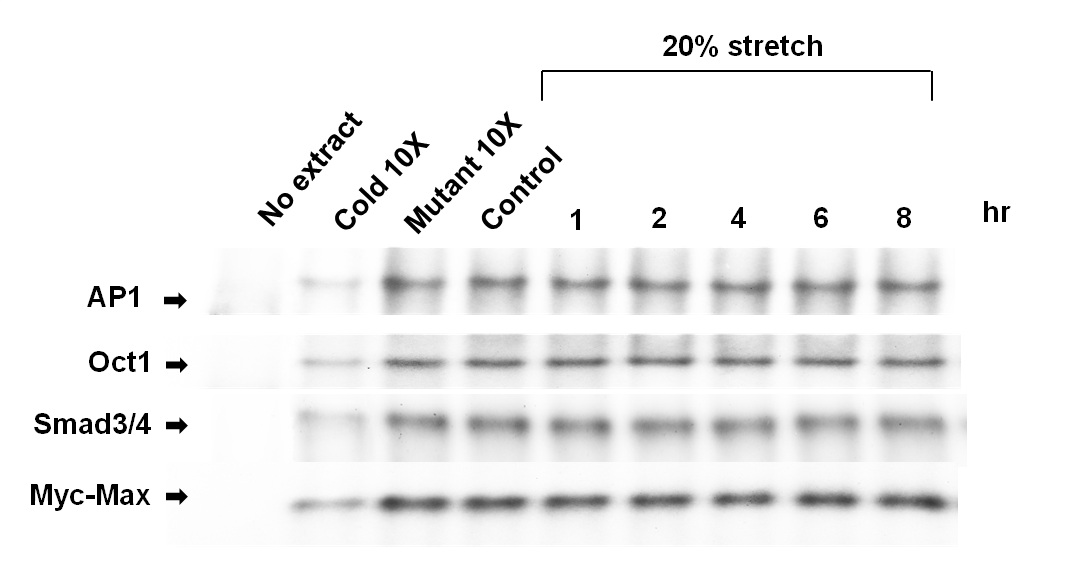

Supplement: S2 Fig — (TIF) [file pone.0148683.s002.tif]

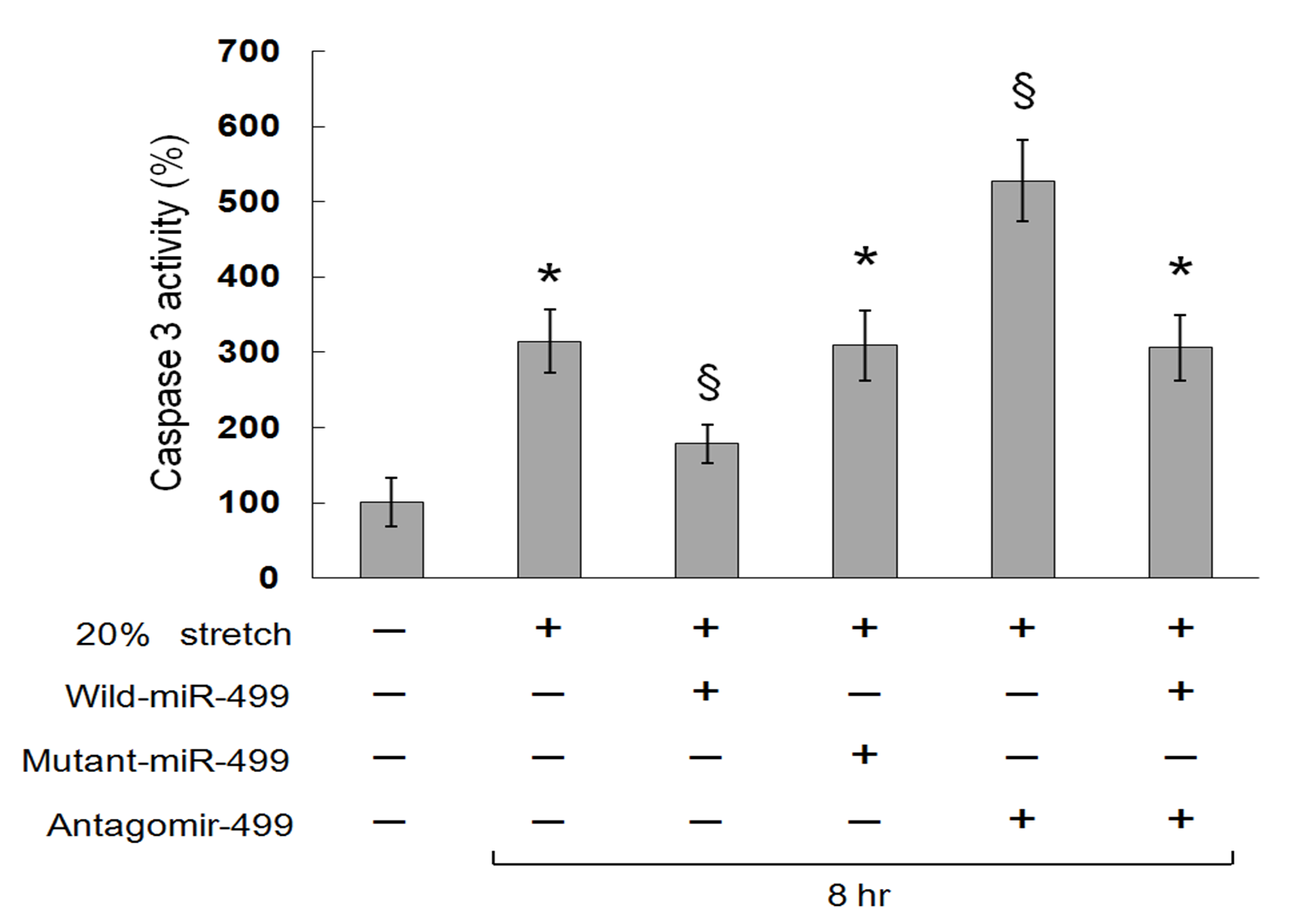

Supplement: S3 Fig — * p < 0.001 vs. control. § p < 0.001 vs. stretch alone. (TIF) [file pone.0148683.s003.tif]

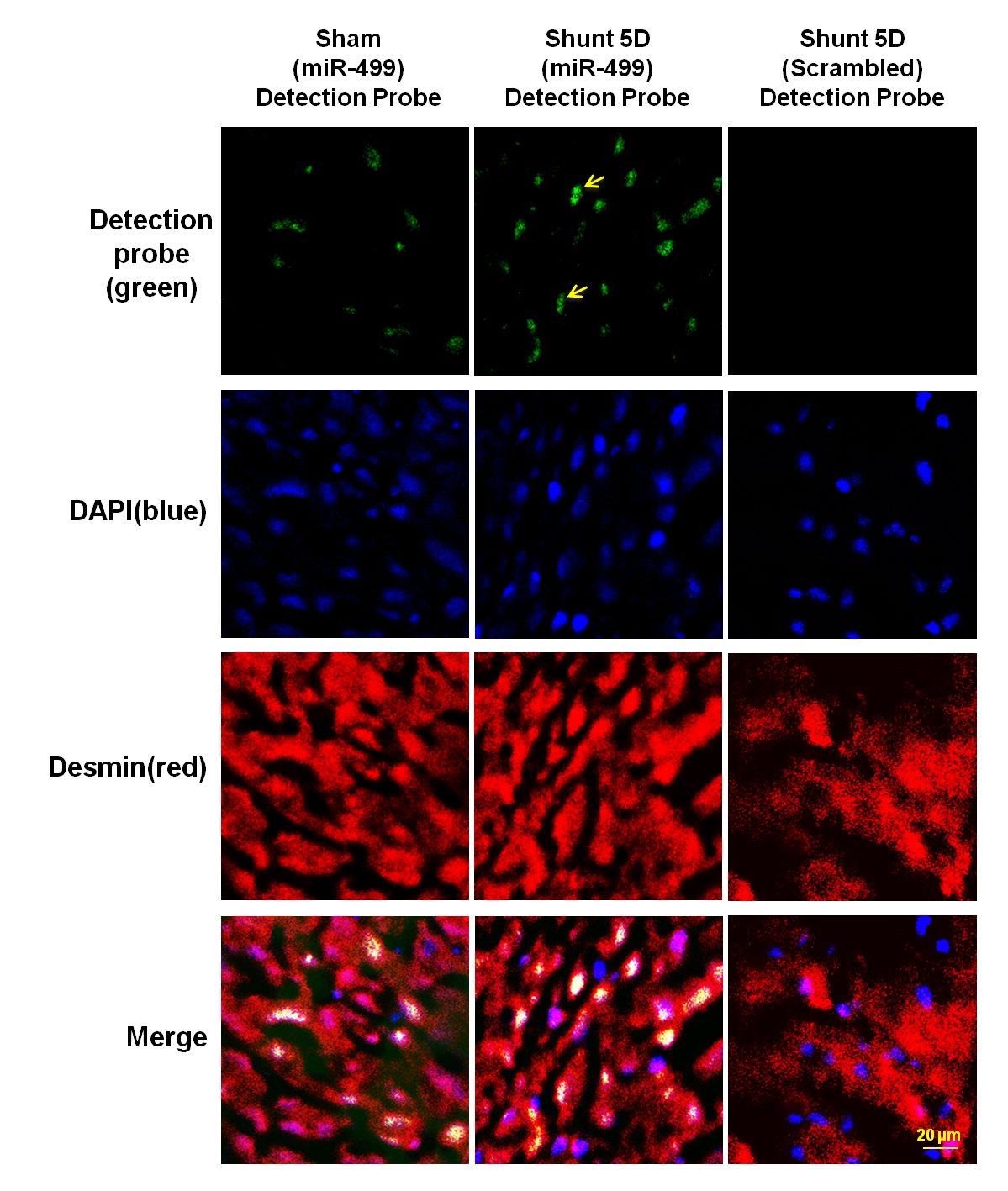

Supplement: S4 Fig — (TIF) [file pone.0148683.s004.tif]

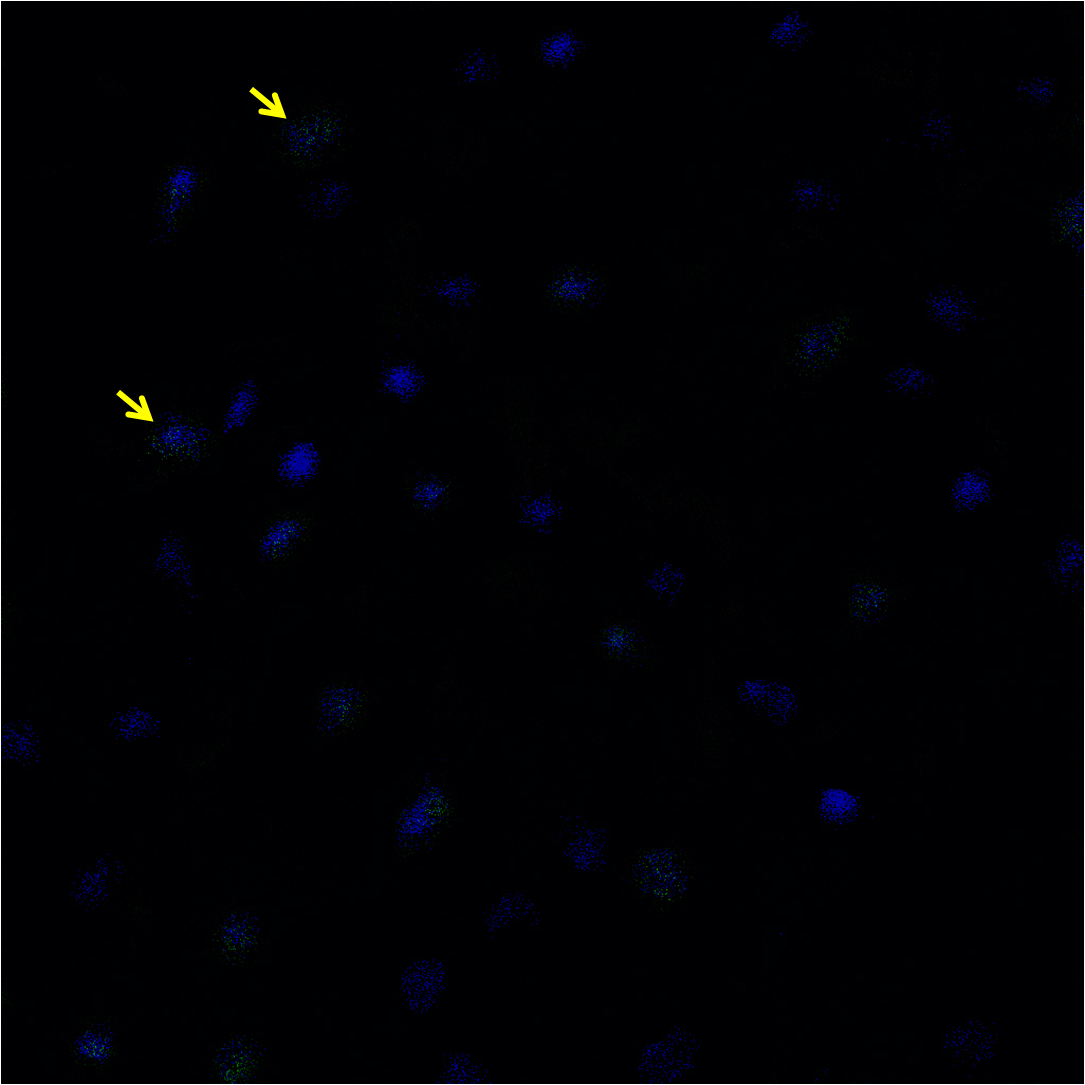

Supplement: S5 Fig — Representative microscopic images showing the presence of miR499 (green color) in the cytoplasm of cardiac myocytes from left ventricular myocardium in AV shunt rats. The sham groups or scrambled probe did not detect the presence of miR499. (TIF) [file pone.0148683.s005.tif]

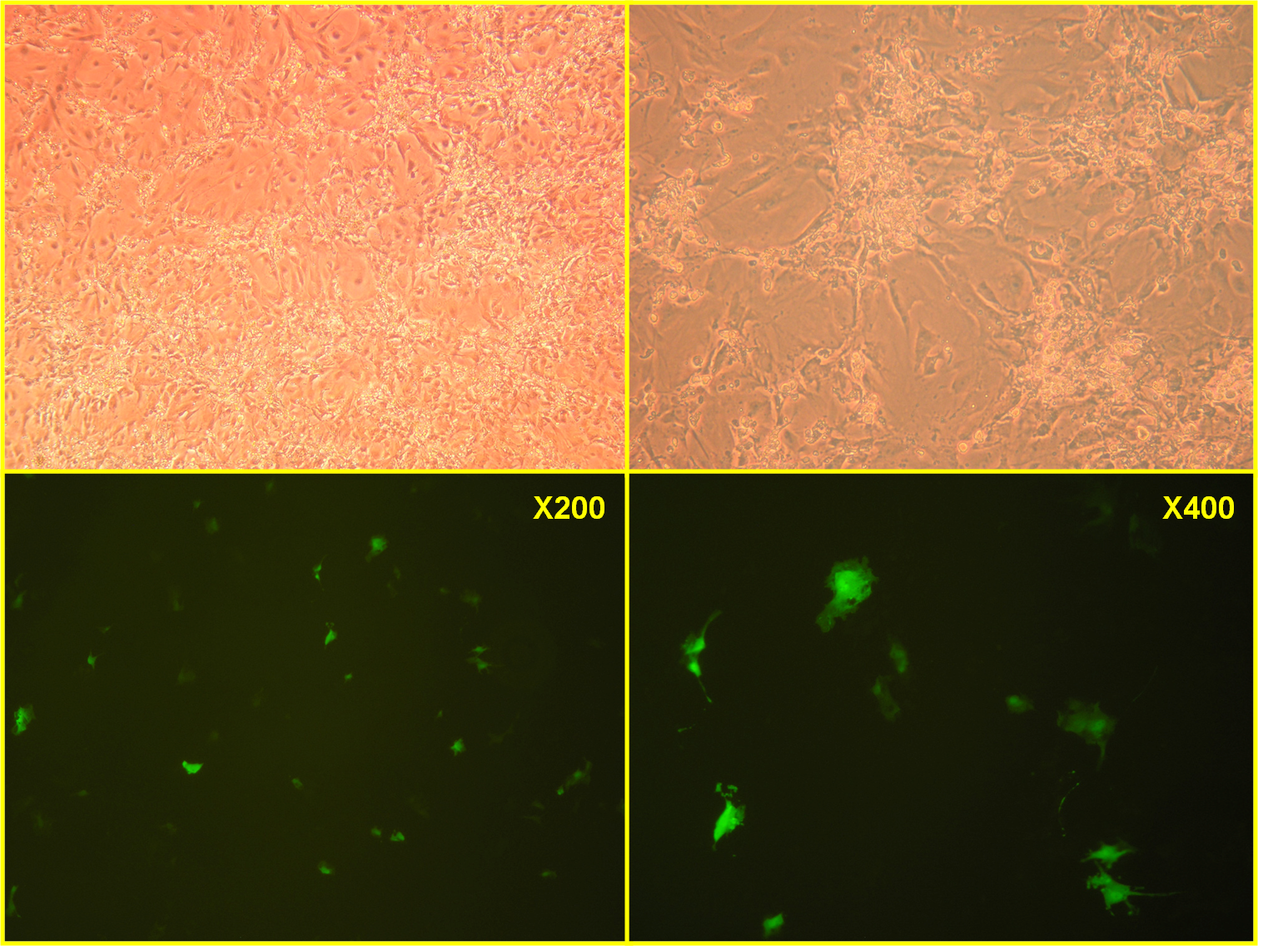

Supplement: S6 Fig — TUNEL staining is indicative of cell death. (TIF) [file pone.0148683.s006.tif]
