# Supplementary material for: Mechanical Stretch Inhibits MicroRNA499 via p53 to Regulate Calcineurin-A Expression in Rat Cardiomyocytes
Source: PLoS One. 2016 Feb 9;11(2):e0148683. doi: 10.1371/journal.pone.0148683 (PMC4747570; doi:10.1371/journal.pone.0148683)
Supplement: S1 File — (DOC) [file pone.0148683.s007.doc]

**Supplement Online Methods**

**The aorta-caval (AV) shunt rat model**

AV shunt was performed on Sprague-Dawley rats weighing between 280g and 330g. On the day of surgery, rats were anesthetized with 2% isoflurane. After confirming a fully anaesthetized state (eg, no response to toe pinch), the vena cava and aorta were exposed via abdominal midline incision. The aorta was punctured at the union of the segment two-thirds caudal to the left renal artery and one-third cephalic to the aortic bifurcation, using an 18 gauge disposable needle held with a plastic syringe. The needle was advanced into the aorta, perforating its adjacent wall and penetrating the vena cava. A bulldog vascular clamp was then placed across the aorta caudal to the left renal artery. After the aorta was clamped, the needle was fully withdrawn and the hole at the aorta was sutured. The clamp was then removed. The patency of the shunt was verified visually by a swelling vena cava and the mixture of arterial and venous blood. Sham-operated control animals were prepared in a similar manner, except that the aorta was not punctured. After the procedure, the abdominal wound was sutured and the rats were allowed to recover. The rats were randomly assigned to experimental or sham groups. The perioperative mortality in the AV shunt group was approximately 10%. All animal procedures were performed in accordance with the Animal Care and Use Committee of Shin Kong Wu Ho-Su Memorial Hospital and conformed with the Guide for the Care and Use of Laboratory Animals as published by the US National Institutes of Health.

**Assessment of cardiac size and function**

Cardiac function of rats in the AV shunt group was evaluated noninvasively on the day of sacrifice by echocardiography (Acuson Sequoia 512 machine; Siemens Medical Solutions, Mountain View, California) using a 15 MHz probe. Left ventricular fractional shortening, left ventricular end-diastolic dimension, and interventricular septum thickness were recorded.

**Real-time Quantitative PCR**

A Lightcycler (Roche Diagnostics, Mannheim, Germany) was used for real-time PCR. cDNA was diluted 1:10 with nuclease-free water. 2 μL of the solution was used for the Lightcycler SYBR-Green mastermix (Roche Diagnostics): 0.5 μmol/L primer, 5 mmol/L magnesium chloride, and 2 μL Master SYBR-Green in nuclease-free water in a final volume of 20 μL. The initial denaturation phase was 5 min at 95 ^o^C followed by an amplification phase (denaturation at 95 ^o^C for 10 sec; annealing at 63 ^o^C for 7 sec; elongation at 72 ^o^C for 8 sec) and detection at 79 ^o^C for 40 cycles. Amplification, fluorescence detection, and post-processing calculation were performed using the Lightcycler apparatus. Individual PCR products were analyzed for DNA sequence to confirm sample purity.

**Quantitative analysis of microRNA**

The reverse transcription reaction was performed using: 10 ng of purified total RNA, 3 mL of miR499 reverse transcription primer (Qiagen Inc., Valencia, CA, USA), 1× reverse transcription buffer (Applied Biosystemsw, Life Technologies, Grand Island, NY, USA), 0.25 mM of each dNTP, 3.33 U/mL Multi-ScribeTM reverse transcriptase (Applied Biosystems), and 0.25U/mL RNase inhibitor (Applied Biosystems). Reactions were incubated in an Applied Biosystems 9700 Thermocycler in a 96-well plate for 30 min at 16° C, 30 min at 42° C, followed by 5 min at 85° C, and then held at 4° C. Each real-time PCR for each miRNA assay (20 mL volume) was carried out in triplicate, and each 20 mL reaction mixture included 1.33 mL of reverse transcription product, 10 mL of 2× TaqMan® Universal PCR Master Mix, and 1 mM 20× TaqMan MicroRNA assay. The reaction was incubated in an Applied Biosystems 7300 Real-Time PCR System in a 96-well plate at 95° C for 10 min, followed by 40 cycles of 95° C for 15 s and 60° C for 1 min. The threshold cycle (C_T_) is defined as the fractional cycle number at which the fluorescence exceeds the fixed threshold of 0.2. The results of quantitative real-time PCR were normalized to that of U6. The sequences of U6 primers were 5'-AACGATACAGAGAAGATTAGCATGGCCC-3'. All fold changes between samples were determined using the comparative C_T_ method.^1^

**Oligonucleotide sequences of Smad3/4, Myc-Max, AP-1, Oct-1 in Electrophoretic mobility shift assay (EMSA)**

Oligonucleotide sequences of Smad3/4 was the consensus 5’-TCGAGAGCCAGACAAAAAGCCAGACATTTAGCCAGACAC-3’. The mutant oligonucleotides sequence was 5’-TCGAGAGCTACATAAAAAGCTACATATTTAGCTACATAC-3’. Myc-Max were the consensus 5’-GGAAGCAGACCACGTGGTCTGCTTCC-3’.The mutant oligonucleotides sequence was 5’- GGAAGCAGACCACGGAGTCTGCTTCC -3’.AP-1 were the consensus 5’-CGCTTGATGACTCAGCCGGAA-3’.The mutant oligonucleotides sequence was 5’- CGCTTGATGACTTGGCCGGAA -3’. Oct-1 were the consensus 5’-TGTCGAATGCAAATCACTAGAA-3’.The mutant oligonucleotides sequence was 5’- TGTCGAATGCAAGCCACTAGAA-3’.

**Western blot analysis**

Cultured cardiomyocytes and tissue samples from left ventricles were homogenized in modified RIPA buffer (50 mmol/L tris [pH 7.4], 1% IGEPAL CA- 630 [Sigma, St. Louis, Missouri], 0.25% sodium deoxycholate, 150 mmol/L NaCl, 1 mmol/L EDTA, 1 mmol/L phenylmethylsulfonyl fluoride, and 1 µg/mL aprotinin, leupeptin, and pepstatin). Equal amounts of protein (30 μg) were loaded into a 10% SDS-polyacrylamide minigel, followed by electrophoresis. Protein samples were mixed with sample buffer, boiled for 10 min, separated by SDS-PAGE under denaturing conditions, and electro blotted to nitrocellulose membranes. The blots were incubated overnight in Tris-buffered saline (TBS) containing 5% milk to block nonspecific binding of the antibody. Rabbit polyclonal anti-CnA antibody, anti-p-Drp1 (phosphorylated form), and anti-Drp1 (unphosphorylated form) antibody (1:200, Santa Cruz Biotechnology, Santa Cruz, USA) were used. Proteins of interest were revealed with specific antibodies as indicated (1:200 dilution) for 1 hour at room temperature, followed by incubation with a 1:10000 dilution of horseradish peroxidase-conjugated polyclonal anti-rabbit antibody for 1 h at room temperature. Signals were visualized using chemiluminenescent detection. Equal protein loading of the samples was verified by staining for the monoclonal antibody α-tubulin. All Western blots were quantified using densitometry.

**Cytotoxicity studies**

Cardiomyocytes were adjusted to 3 × 10^4^ cells/ml in growth medium. Aliquots (20ml) of cell suspension were plated in 40-mm Petri dishes. After incubation for 24 h, the medium was replaced with fresh medium containing interesting monoclonal antibody. After incubation for 24 h, the medium was aspirated off and 0.5 mg/ml MTT solution was added and incubation continued for another 4 h. At the end of the incubation period, the medium was removed from the attached cells and the converted dye crystals were dissolved with DMSO. Absorbency of converted dye was measured at a wavelength of 570 nm. For detection of cell injury induced by stretch, cell viability after application of cyclic stretch was monitored by trypan blue staining.

**Terminal deoxynucleotidyl transferase-mediated dUTP nick-end labeling (TUNEL) assay**

DNA fragmentation was determined using a TUNEL assay with ApopTag peroxidase in situ apoptosis detection kit (Chemicon International, Temecula, CA). Left ventricular myocardium was fixed in 4% paraformaldehyde for 10 min followed by a staining procedure according to manufacturer’s protocol. In this method, DNA strand breaks were detected using enzymatically labeling the free 3’-OH termini with modified nucleotides with terminal deoxynucleotidyl transferase (TdT). DNA fragments were then labeled with digoxigenin and bound to anti-digoxigenin antibody conjugated to horseradish peroxidase. The peroxidase conjugate produces a localized stain which can be detected. Fluorescence signals were obtained with a confocal microscope (Nikon D-ECLIPSE) and assayed using its associated image processing and analysis software.

**Immunohistochemical analysis**

Rat left ventricles were harvested and fixed in 10% formaldehyde and sliced into 5 mm sections and embedded in paraffin. For immunohistochemical analysis, the slides were postfixed in 4% paraformaldehyde for 20 min, treated in 3% hydrogen peroxide/PBS for 25 min, blocked in 5% normal rabbit serum for 20 min, blocked with biotin/avidin for 15 min each, and incubated with fluorescent isothiocyanate (FITC)-conjugated rabbit monoclonal anti-CnA antibody (Santa Cruz Biotechnology, Santa Cruz, USA) for 2 hours at room temperature. Next samples were incubated with biotinylated rabbit-anti mouse IgG at 1:400 for 30 min, and Vector Elite ABC biotin-avidin-peroxidase complex for 30 min. Sections were then developed with diaminobenzidine and diaminobenzidine enhancer (Vector), counterstained with hematoxylin. Images were examined using a fluorescent microscope.

**In situ hybridization assay**

Five-micrometer-thick tissue sections of left ventricular myocardium were mounted on positively charged barrier frame slides, dewaxed in xylenes, and rehydrated through an ethanol dilution series (100% to 25%). Tissue sections were digested with 5 mg/mL of proteinase K for 20 minutes at 37ºC to facilitate probe penetration and exposure of miRNA species. To minimize nonspecific binding based on charge interactions, tissues were subjected to a brief acetylation reaction [66 mmol/L HCl, 0.66% acetic anhydride (v/v) and 1.5% triethanolamine (v/v) in RNasefree water]. Then, tissue sections were prehybridized at the hybridization temperature for 30 minutes in prehybridization solution which consisted of 50% deionized formamide, 5 X sodium chloride/sodium citrate buffer, 1 x Denhardt’s solution, 500 mg/mL of yeast tRNA, and 0.01% Tween. The prehybridization solution was replaced with 200 ml of hybridization solution containing 10 pmol of the FAM-labeled LNA miR-499 probe (product sequence 5’-3’, AGCACAGACTTGCTGTGATGTT, Exiqon, Vedbaek, Denmark) and tissues were incubated for 90 minutes at the hybridization temperature and washed twice for 10 minutes in sodium chloride/sodium citrate buffer.

**Reference**

1. Schmittgen TD, Livak KJ. Analyzing real-time PCR data by the comparative C(T) method. Nat Protoc 2008;3:1101-1108.
