# Supplementary material for: Mechanical Stretch Inhibits MicroRNA499 via p53 to Regulate Calcineurin-A Expression in Rat Cardiomyocytes
Source: PLoS One. 2016 Feb 9;11(2):e0148683. doi: 10.1371/journal.pone.0148683 (PMC4747570; doi:10.1371/journal.pone.0148683)
Supplement: S1 Table — (DOC) [file pone.0148683.s008.doc]

Supplementary Table 1. Hemodynamic and echocardiographic parameters of the failing heart induced by aorta-caval shunt

|  | Sham | Shunt 7D | Shunt 14D | Shunt 28D | Shunt 14D + miR-499 | Shunt 14D + miR-499 + Antagomir-499 | Shunt 14D + Mut-499 |
| --- | --- | --- | --- | --- | --- | --- | --- |
| N | 3 | 4 | 3 | 3 | 4 | 3 | 3 |
| Body weight, g | 307 ± 18 | 314 ± 26 | 304 ± 30 | 302 ± 32 | 309 ± 15 | 299 ± 27 | 307 ± 16 |
| Heart weight, mg | 812 ± 47 | 912 ± 51 | 1129 ± 66* | 1183 ± 69* | 988 ± 54^+^ | 1082 ± 42* | 1091 ± 37* |
| Heart weight/ body weight, mg/g | 2.4± 0.5 | 2.8 ± 0.7 | 3.43 ± 0.8* | 3.53 ± 0.6* | 3.0 ± 0.8^+^ | 3.45 ± 0.6* | 3.4 ± 0.5* |
| Heart rate, min | 315 ± 27 | 357 ± 22 | 300± 28 | 310 ± 36 | 326 ± 38 | 309 ± 29 | 312 ± 21 |
| MAP, mmHg | 84 ± 12 | 74 ± 10 | 68 ± 7 | 63 ± 8 | 72 ± 8 | 70 ± 9 | 71 ± 12 |
| IVSTd, mm | 1.4 ± 0.3 | 1.3 ± 0.6 | 1.2 ± 0.9 | 1.0 ± 0.6 | 1.3 ± 0.6 | 1.1 ± 0.4 | 1.2 ± 0.4 |
| LVPWT, mm | 1.2 ± 0.4 | 1.2 ± 0.3 | 1.1 ± 0.7 | 1.0 ± 0.4 | 1.3 ± 0.4 | 1.2 ± 0.3 | 1.1 ± 0.6 |
| LVEDD, mm | 6.4 ± 0.3 | 6.8 ± 0.7 | 7.2 ± 0.4* | 7.3 ± 0.7* | 6.6 ± 0.6^+^ | 7.0 ± 0.5* | 7.1 ± 0.7* |
| LVESD, mm | 3.3 ± 0.5 | 3.7 ± 0.5 | 4.0 ± 0.5* | 4.3 ± 0.5* | 3.7 ± 0.8^+^ | 3.9 ± 0.6* | 4.0 ± 0.8* |
| FS, % | 48 ± 7 | 44 ± 9 | 41 ± 11 | 39 ± 9 | 44 ± 8 | 41 ± 7 | 42 ± 9 |
| MAP =mean arterial pressure. IVSTd = inter-ventricular septum end-diastolic thickness. LVPWT = left ventricular posterior wall thickness. LVEDD = left ventricular end-diastolic dimension. LVESD = left ventricular end-systolic dimension. FS = fraction shortening. *p < 0.01 vs. sham, + p < 0.01 vs. shunt 14D | | | | | | | |
